# Reliability and suitability of physiological exercise response and recovery markers

**DOI:** 10.1038/s41598-020-69280-9

**Published:** 2020-07-17

**Authors:** Thomas Reichel, Tim K. Boßlau, Jana Palmowski, Klaus Eder, Robert Ringseis, Frank C. Mooren, Rüdiger Walscheid, Evita Bothur, Stefan Samel, Torsten Frech, Marc Philippe, Karsten Krüger

**Affiliations:** 1 Institute of Sports Science, Department of Exercise and Health, Leibniz University Hanover, 30167 Hannover, Germany; 2 Department of Exercise Physiology and Sports Therapy, Institute of Sports Science, Justus-Liebig-University Giessen, Kugelberg 62, 35394 Giessen, Germany; 3 Institute of Animal Nutrition and Nutrition Physiology, Justus-Liebig-University Giessen, 35394 Giessen, Germany; 4 Department of Rehabilitation, Faculty of Health, Witten/Herdecke University, 58455 Witten, Germany; 5 Medical Center for Laboratory Medicine and Microbiology, Koblenz-Mittelrhein, 56068 Koblenz, Germany

**Keywords:** Physiology, Biomarkers

## Abstract

There is currently insufficient evidence about the reliable quantification of exercise load and athlete’s recovery management for monitoring training processes. Therefore, this test–retest study investigated the reliability of various subjective, muscle force, and blood-based parameters in order to evaluate their suitability for monitoring exercise and recovery cycles. 62 subjects completed two identical 60-min continuous endurance exercise bouts intermitted by a four-week recovery period. Before, immediately after, three, and 24 h after each exercise bout, analysis of parameters were performed. Significant changes over time were found for rating of perceived exertion (RPE), multidimensional mood state questionnaire (MDMQ), maximum voluntary contraction parameters (MVCs), and blood-based biomarkers (*p* < 0.05). Excellent reliability was calculated for MVCs, mean corpuscular volume and 5-bound distance (ICC > 0.90). A good reliability was found for thiobarbituric acid reactive substances (TBARS) (ICC = 0.79) and haematological markers (ICC = 0.75–0.86). For RPE, MDMQ, interleukin (IL-) 1RA, IL-6, IL-8, IL-15, cortisol, lactate dehydrogenase (LDH), creatine kinase (CK) only moderate reliability was found (ICC < 0.75). Significant associations for IL1-RA and CK to MVC were found. The excellent to moderate reliability of TBARS, LDH, IL-1RA, six measured haematological markers, MVCs and MDMQ implicate their suitability as physiological exercise response and recovery markers for monitoring athletes’ load management.

## Introduction

The reliable quantification of individuals’ physiological response to acute exercise bouts are of major importance for monitoring training. Both, subjective as well as objective markers are used to control athletes’ training in accordance with individual abilities. When training stimuli are wrongly applied due to a lack in load management, it results in an imbalance in the exercise load-recovery cycle with the well-known consequence of overtraining, increased risk of injury, and the inhibition of fundamental adaptation processes^1–3^. Therefore, it seems desirable to further explore the reliability of subjective and objective markers in order to understand how individual athletes deal with physical strains, and thus optimize exercise load-recovery balance. The need of accurate and reproducibly biomarkers in clinical practice, which reflect objective, quantifiable medical signs or effects of treatments and interventions in physiology of biochemical processes, has become a commonplace character^4^.

In order to evaluate and establish suitable markers for monitoring training, and recovery, it is important to investigate subjective and objective process-based changes with high levels of accuracy and precision^5^. However, these requirements are questionable for various parameters, since many biomarkers have crucial limitations and an immense fluctuation width^1–3^. Previous studies concluded that it is important to assess the reliability and specificity of markers in controlled interventional trials^6^. Particularly in the context of sport, there are only limited data in consideration of reliable markers which reflect the physiological exercise response and recovery processes^7^.

Actually, there is still insufficient knowledge available as to which specific parameters are most suitable to monitor exercise load-recovery status^1^. Some metabolic, immunological and haematological markers are commonly used as objective physiological exercise response indicators^8^. However, markers such as creatine kinase (CK), which is released in response to muscle fiber damage, are known to have a large intraindividual and interindividual variability^9,10^. Though, the detailed evaluation of selected haematological parameters, inflammatory markers, enzymes, and metabolic markers such as haemoglobin (HGB)^8^, interleukin (IL-)-1 receptor antagonist (IL-1RA)^11^, lactate dehydrogenase (LDH)^12^ or thiobarbituric acids (TBARS)^13^ are still pending. These markers were chosen due to their use in exercise context analyzing immune activation, metabolic demands, or oxidative stress^14^. Besides, regarding a comprehensive and serious assessment of athletes’ load state, the use of combinations of suitable parameters including functional testing, subjective testing and biochemical analyses should be considered^15,16^. In this regard, suitability is defined by a certain exercise sensitivity and correlation to muscle force parameters, such as maximum voluntary contraction (MVC)^17^. Some studies proved the suitability of muscle force and subjective parameters such as the rating of perceived exertion (RPE) or the multidimensional mood state as subjective tools for physical performance assessments^18–20^. Accordingly, the discovery of additional markers might contribute to creating a panel of parameters which might offer the possibility of analyzing multiple aspects of human performance and health status.

The aim of the current study was to examine the different exercise response, changes during recovery, and the test–retest reliability of various subjective parameters, muscle force values and blood biomarkers after strenuous bouts of endurance exercise; thereby evaluating their suitability for monitoring exercise load and recovery. We hypothesized that some of the analyzed parameters are suitable and reliable as markers which reflect the physiological exercise response and recovery processes and can be used for sports practice.

## Results

### Blood biomarkers

**Figure 1:**
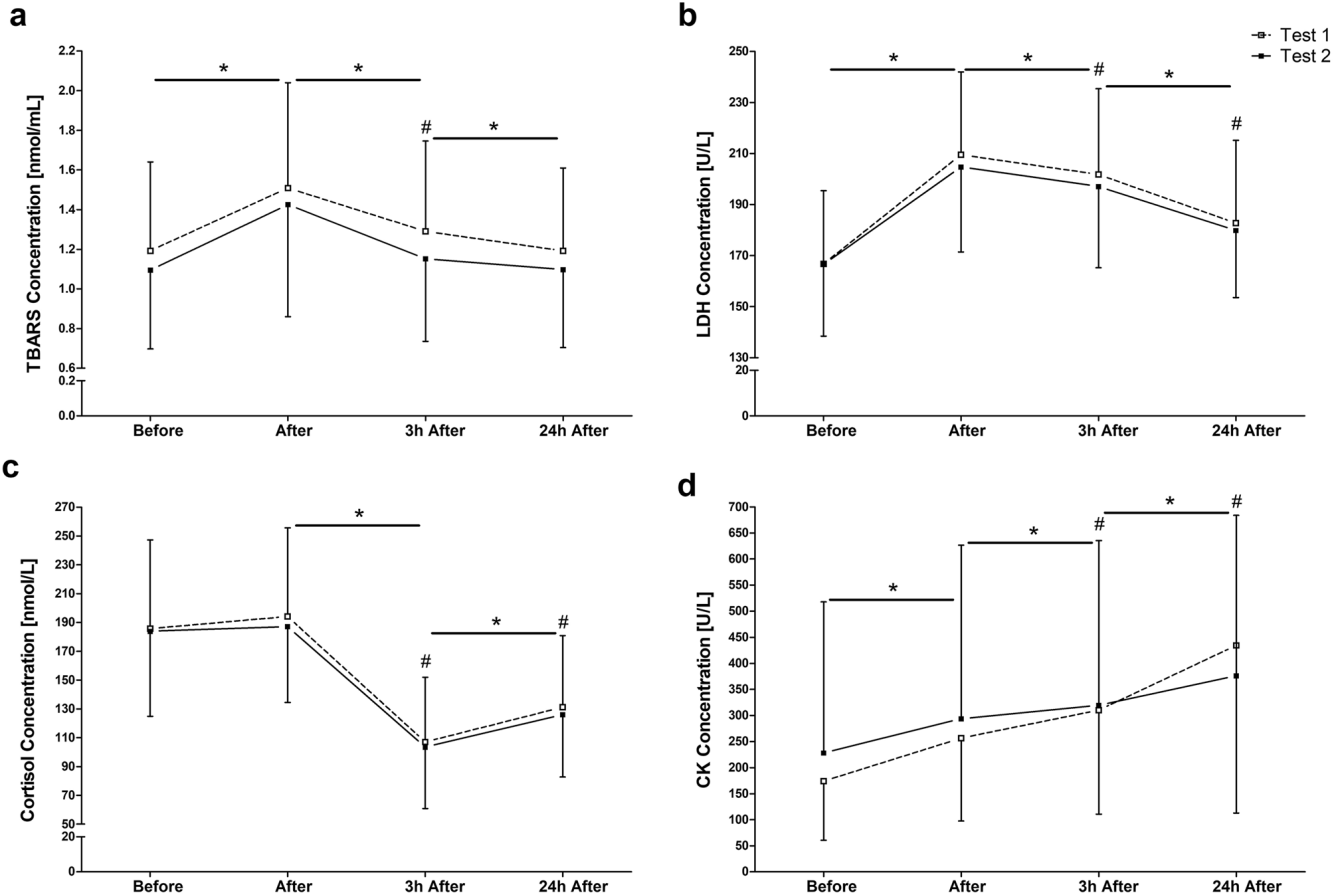
Changes of metabolites (**a**), enzymes (**b**, **d**), and hormones (**c**) after, 3 h after and 24 h after two identical endurance running field tests (Test 1 and Test 2). Data are presented as mean ± standard deviation. *Significantly different from previous measuring time point of both tests. #Difference against pre-exercise (before) in both tests (*p* < 0.05).

Results indicated that TBARS (*F*_3_ = 79.43; *p* < 0.001), LDH (*F*_3_ = 208.72; *p* < 0.001), CK (*F*_1.44_ = 153.09; *p* < 0.001) and cortisol (*F*_2.33_ = 99.89; *p* < 0.001) showed significant main effects over the measuring time points (MTPs). Both exercise trials induced an increase of TBARS (Fig. 1a), LDH (Fig. 1b) and CK (Fig. 1d) immediately after the tests (*p* < 0.001), whereas no changes were found for cortisol (Fig. 1c). While the concentrations of TBARS, LDH and cortisol decreased three hours after the running field tests (RFTs) (*p* < 0.001), CK further increased (*p* < 0.001). Nevertheless, all four biomarkers showed significant differences 3 h (3 h) after the exercise tests compared to pre-exercise levels (*p* < 0.001). 24 h (24 h) after, levels of TBARS decreased back to baseline (3–24 h, *p* = 0.018). In contrast, concentrations of CK further increased (3–24 h, *p* < 0.001), while cortisol concentration turned again into a rise (3–24 h, *p* < 0.001). LDH, CK and cortisol were still increased after 24 h compared with pre-exercise levels (*p* < 0.001). Analysis of reliability exhibited good to moderate intraclass correlation coefficients (ICCs) for TBARS (ICC = 0.79), LDH (ICC = 0.73), cortisol (ICC = 0.7) and CK (ICC = 0.64).

**Figure 2:**
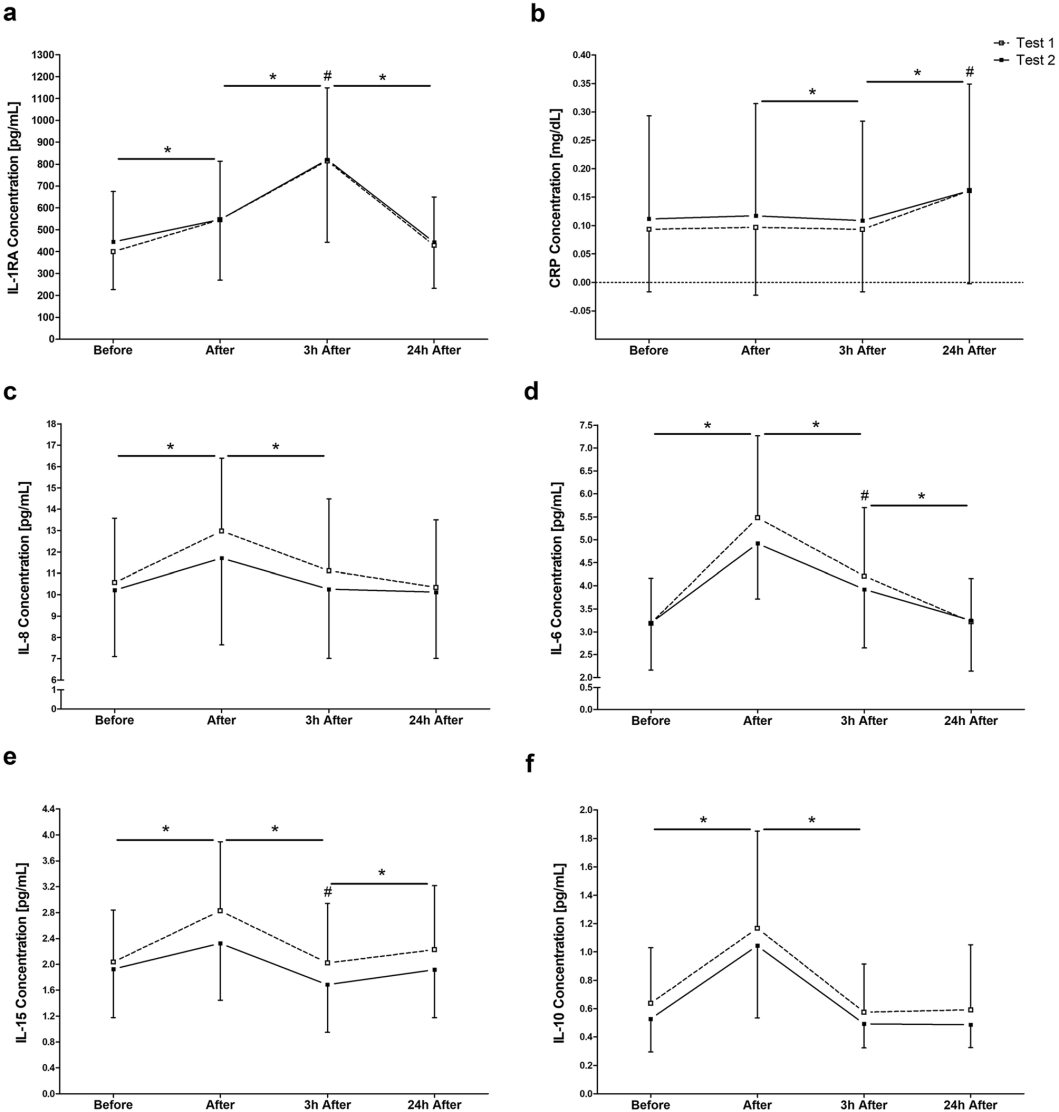
Concentrations of various cytokines (**a**, **c**, **d**, **e**, **f**) and plasma proteins (**b**) before, after, 3 h after and 24 h after two identical endurance running field tests (Test 1 and Test 2). Data are presented as mean ± standard deviation. *Significantly different from previous measuring time point of both tests. #Difference against pre-exercise (before) in both tests (*p* < 0.05).

Significant main effects were found for the cytokines IL-1RA (*F*_2.36_ = 210.85; *p* < 0.001), IL-8 (*F*_3_ = 30.09; *p* < 0.001) and C-reactive protein (CRP) (*F*_1.39_ = 63.52; *p* < 0.001) over the measuring time points. The RFTs induced an increase in IL-1RA, IL-6, IL-8, IL-10, and IL-15 immediately after exercise (*p* < 0.0001). In contrast, no immediate changes of CRP levels (Fig. 2b) were found. Peaking immediately after, concentrations of IL-8 (Fig. 2c), IL-6 (Fig. 2d), IL-15 (Fig. 2e), and IL-10 (Fig. 2f) decreased 3 h after the RFTs (*p* < 0.001), while IL-6 and IL-15 were still increased compared to pre-exercise values (IL-6: *p* < 0.001; IL-15: *p* = 0.004). Similarly, IL-1RA (Fig. 2a) peaked 3 h after the RFTs (*p* < 0.001). Levels of both, IL-6 and IL-1RA, returned to baseline at 24 h after the running trials (*p* < 0.001). CRP showed a delayed peak at 24 h after the RFTs. For IL-15 a further increase was found between 3 and 24 h after the RFTs (*p* < 0.0001). The best test–retest reliability was measured for IL-1RA (ICC = 0.72). Moderate ICC values were calculated for CRP (ICC = 0.71), IL-8 (ICC = 0.67), IL-6 (ICC = 0.64) and IL-15 (ICC = 0.55), while the ICC of IL-10 was below 0.5 (ICC = 0.24).

**Figure 3:**
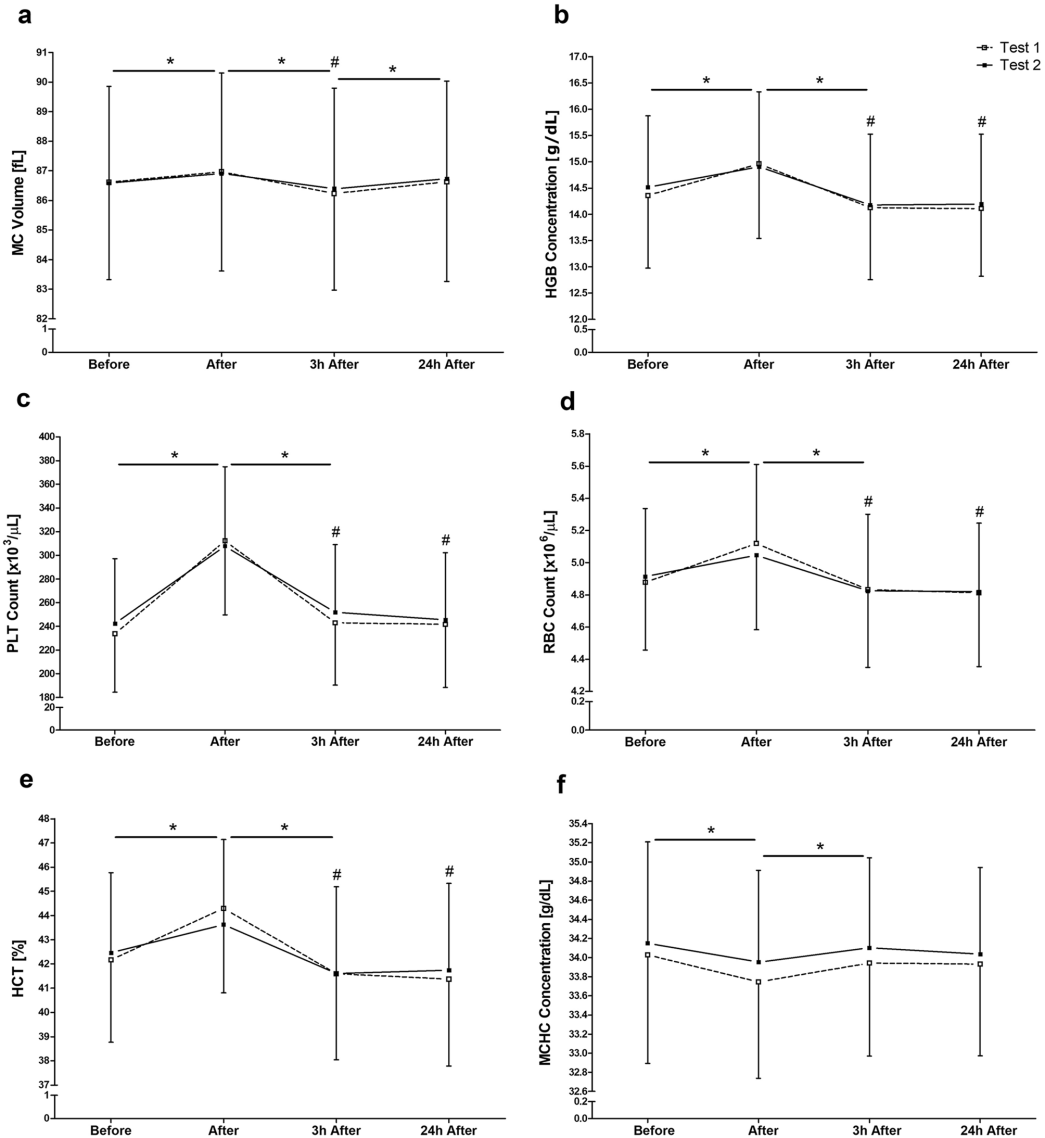
Effects of both running field tests (Test 1 and Test 2) on various haematological markers after, 3 h after and 24 h after. Data are presented as mean ± standard deviation. *Significantly different from previous measuring time point of both tests. #Difference against pre-exercise (before) in both tests (*p* < 0.05).

For all blood cell related markers, the two-way ANOVA revealed significant differences over the MTPs, such as mean corpuscular volume (MCV) (*F*_3_ = 56,15; *p* < 0.001) and platelets (PLT) (*F*_2.48_ = 342.75; *p* < 0.001). Post hoc analysis of blood cell related markers showed an increase in MCV, haemoglobin (HGB), PLT, erythrocytes (RBC) and haematocrit (HCT) (Fig. 3a–e) immediately after exercise (*p* < 0.001). In contrast, exercise trials were followed by a decrease of mean corpuscular haemoglobin concentration (MCHC) (Fig. 3f) immediately after the RFTs (*p* = 0.005). While MCV, PLT, HGB, RBC and HCT decreased 3 h after (*p* < 0.001), the MCHC values turned into a significant increase (*p* < 0.001). The concentration of MCV increased (*p* < 0.001) 24 h after both RFTs, again. For HGB, PLT, RBC, and HCT significant differences between pre-exercise and 3 h (*p* < 0.001) and 24 h (HGB: *p* < 0.001; HCT: *p* < 0.002; PLT: *p* < 0.046; RBC: *p* < 0.001) were shown. With regard to the test–retest reliability, excellent to good ICC levels were found for MCV (ICC = 0.96), HGB (ICC = 0.86), PLT (ICC = 0.86), RBC (ICC = 0.84), HCT (ICC = 0.78) and MCHC (ICC = 0.75). However, for other blood markers such as mean corpuscular haemoglobin (MCH), lymphocytes (LYM), platelets-lymphocyte ratio (PLR), neutrophils (NEU), neutrophils-lymphocytes ratio (NLR), systemic immune-inflammation index (SII) and leukocytes (WBC), no significant changes over the MTPs or only moderate ICC values between 0.51 – 0.74 were found (data not shown).

### Muscle force parameters

**Figure 4:**
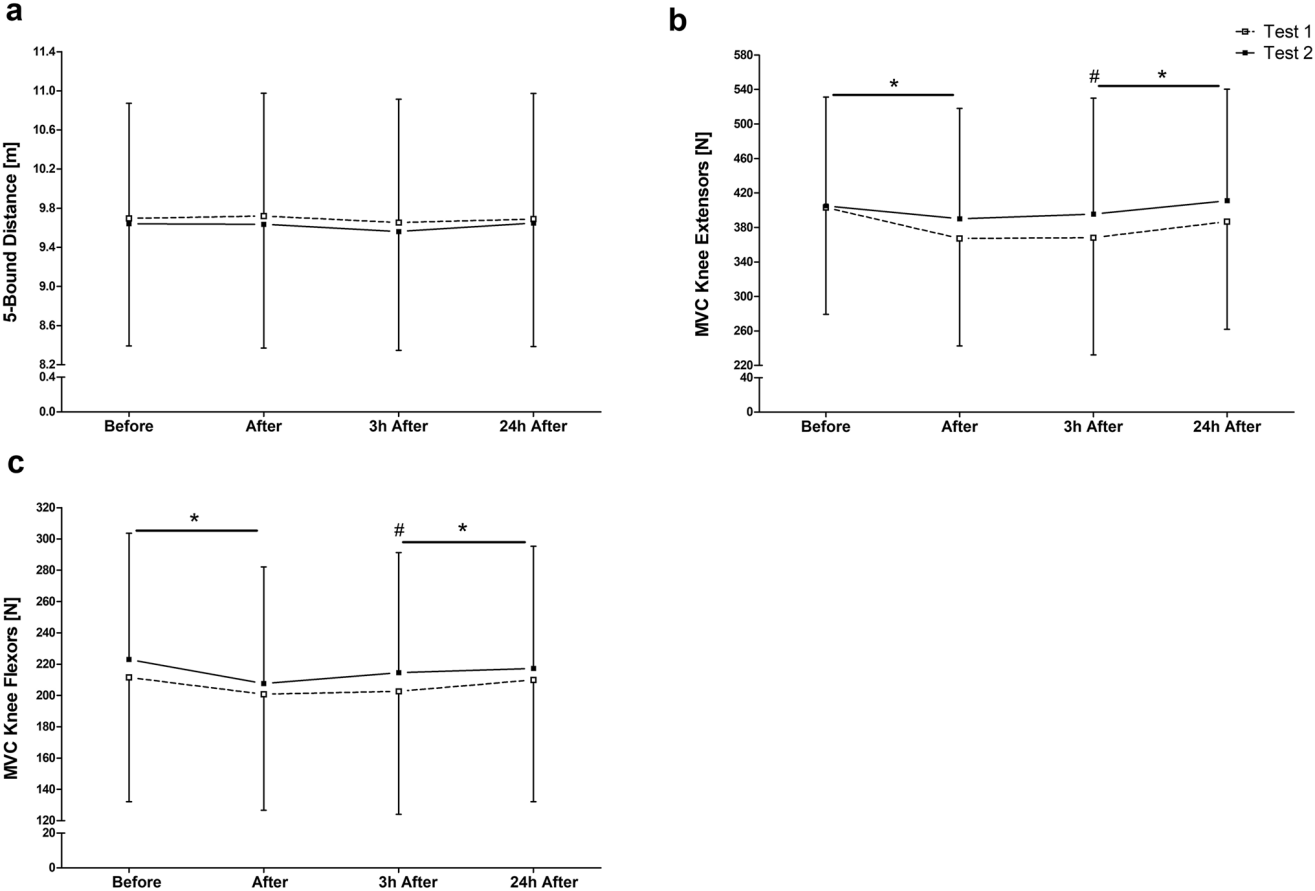
Five-bound test for jump distance (**a**), MVC in knee extensors (**b**), and knee flexors (**c**) for muscle force before, after, 3 h after and 24 h after two identical endurance running field tests (Test 1 and Test 2). Data are presented as mean ± standard deviation. *Significantly different from previous measuring time point of both tests. #Difference against pre-exercise (before) in both tests (*p* < 0.05).

No changes over time were found for the 5-bound distance (Fig. 4a). However, for this test an excellent ICC of 0.96 was calculated. The two-way ANOVA revealed significant differences in both MVCs (knee flexion: *F*_2.61_ = 14.18, *p* < 0.001; knee extension: *F*_2.49_ = 25.01, *p* < 0.001) parameters over the MTPs. MVCs of the knee extensors (Fig. 4b) and the knee flexors (Fig. 4c) decreased immediately after the RFTs (*p* < 0.001). Both parameters returned to pre-exercise values at 3 h after exercise. Excellent ICC values for the MVC of knee extensors (ICC = 0.93), as well as knee flexors (ICC = 0.92) were calculated.

### Subjective parameters

**Figure 5:**
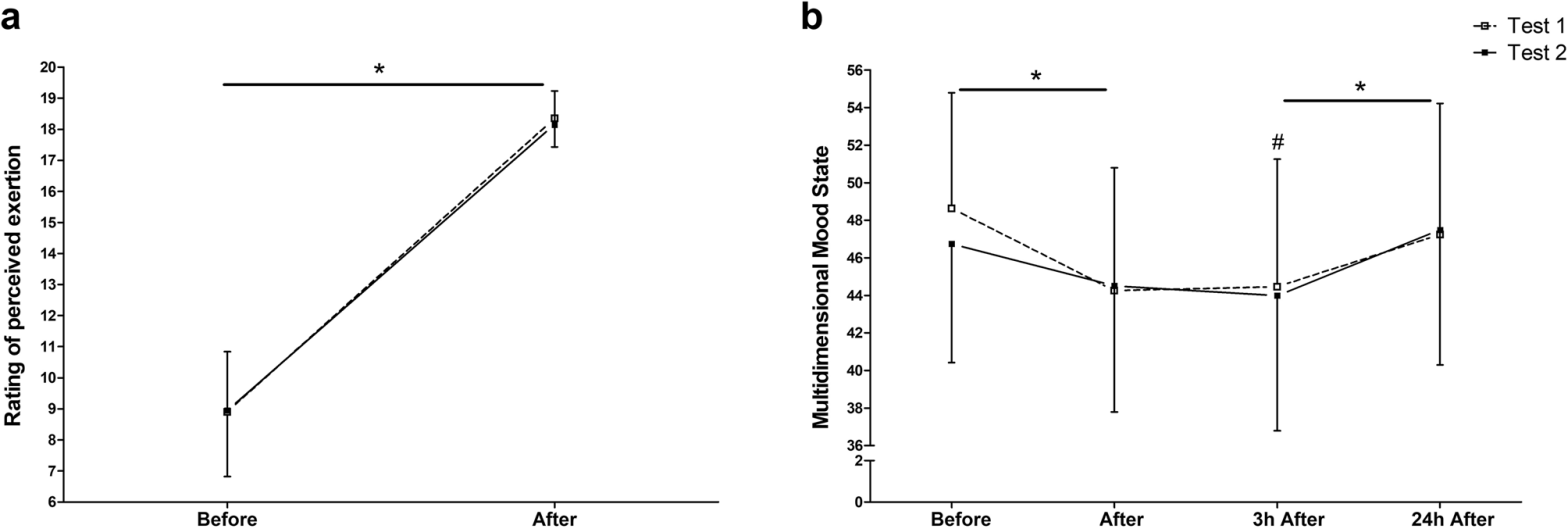
Rating of perceived exertion (**a**) requested using BS and multidimensional mood state (**b**) scaling by using the MDMQ before, after, 3 h after and 24 h after two identical endurance running field tests (Test 1 and Test 2). Data are presented as mean ± standard deviation. *Significantly different from previous measuring time point of both tests. #Difference against pre-exercise (before) in both tests (*p* < 0.05).

The two-way ANOVA revealed significant changes over time for RPE values (Fig. 5a) (*F*_3_ = 345.19; *p* < 0.001). For the test–retest reliability, a poor ICC of 0.49 was found. Data from the acute multidimensional mood state showed lower scores immediately after the RFTs as well as three hours after exercise. We compared this to pre-exercise levels (*F*_2.62_ = 13.94; *p* < 0.001) (Fig. 5b). Values increased back to baseline between three hours and 24 h after (*p* = 0.001). In view of the results between both MTPs, an ICC of 0.70 was found.

### Correlation analysis

A correlation was found between MVC in knee extension and TBARS immediately after (*r* = 0.26, *p* = 0.044), 3 h after (*r* = 0.34, *p* = 0.009) and 24 h after (*r* = 0.30, *p* = 0.020) the RFT at testing day 1 (TD1). Interestingly, there were also correlations in the changes between the pre-exercise value and 3 h after (*r* = -0.30, *p* = 0.021). In addition, the MVC in knee flexion correlated with the TBARS three hours after (*r* = 0.31, *p* = 0.031) and 24 h after (*r* = 0.28, *p* = 0.030). In confirmation with these results, the differences in measuring times pre-exercise to 24 h after could also correlate between knee flexion and TBARS (*r* = −0.26, *p* = 0.048) An association between CK and MVC knee extension over all MTPs (*r* = 0.40, *p* = 0.002; *r* = 0.38, *p* = 0.004; *r* = 0.28, *p* = 0.034; *r* = 0.36, *p* = 0.006) was detected. Furthermore, correlations between the MVC values in knee flexion and CK was determined based on the calculations between the changes from pre- to post-exercise (*r* = 0.27, *p* = 0.039) as well as to 3 h after (*r* = -0.36. *p* = 0.005). Interestingly, correlation analysis revealed an association between both MVC parameters and IL-1RA immediately after exercise at TD1 (MVC knee flexors—IL-1RA: *r* = 0.31, *p* = 0.017; MVC knee extensors—IL-1RA: *r* = 0.31, *p* = 0.019). Finally, a significant correlation of the changes between IL-1RA and MVCs pre- and post-exercise was found (*r* = 0.27, *p* < 0.044). The trend of the MVC values was similar to the trend of all correlated biomarkers.

### Effects of training status

No differences between trained and untrained participants were found for the changes of any parameter over time. With regard to the reliability, CK revealed a poor reliability in the subgroup of trained individuals (ICC = 0.49), while reliability was categorized as good (ICC = 0.84) for the untrained group. The opposite was true for CRP. In the subgroup of trained participants a good reliability was found (ICC = 0.80), while the reliability in the untrained subgroup was calculated as poor (ICC = 0.48).

## Discussion

The novel findings of the present study are the high reliability of TBARS, LDH, IL-1RA, MCV, HGB, PLT, RBC, HCT, and MCHC after two identical controlled bouts of endurance exercise, suggesting their suitability as blood-based biomarkers for monitoring physiological exercise response and recovery status in endurance athletes. Based on the high associations to MVC, CK seems to be a suitable biomarker. However, the reliability of CK was only moderate, questions its use as a marker in sports practice. MVCs and the MDMQ seem to represent appropriate complementary monitoring tools to the blood markers.

Regarding blood-based biomarkers, the highest reliability was found for TBARS, followed by LDH and IL-1RA values. A similar physiological exercise response-recovery curve for the TBARS concentrations was shown in a recent study by Krüger et al. (2016) after a 30 min continuous bicycle test^21^. Other findings confirm a high responsiveness of TBARS after exercise, which physiologically reflects an increased oxidative stress and subsequently an enhanced lipid peroxidation after acute exercise^22^. The observed correlations between TBARS and MVCs after the endurance exercise support an association between levels of oxidative stress and muscle force, and also proved the potential suitability of TBARS as a diagnostic parameter in endurance sports^2,10^. Despite the observed high reliability of TBARS as a parameter for evaluating physiological exercise response and recovery processes, the method of TBARS analysis has been generally considering critically in the literature due to several limitations. As discussed by Cobley et al. (2017), a major flaw of TBARS measurement is the low specificity of TBARS which react with various substrates to form malondialdehyde (MDA) such that most MDA is generated by the assay itself^23^. In addition, the heating step during TBARS analysis causes partial lipid decomposition leading to the formation of extraneous MDA. However, current data proved that standardized procedures might compensate for some methodological flaws. LDH values showed similar kinetics like TBARS over time. With regard to high reliability of blood LDH values and results of previous studies, this marker might represent another suitable biomarker for exercise load and recovery^24^. Within the cytokines, IL-1RA showed the highest reliability and, in parallel, seems to be associated with physiological exercise response and progressive recovery. IL-1RA is secreted by various types including immune cells, and inhibits pro-inflammatory activities of various cytokines. The observed changes in IL-1RA concentration correspond with earlier observations which showed a peak at one and a half to two hours after exercise and a decrease back to baseline levels 24 h after treadmill running^25^. However, the reliability of IL-1RA has not been established so far. Interestingly, we found an association to MVC, suggesting a link to muscular fatigue. Thus, the anti-inflammatory cytokine, IL-1RA, seems to be a reliable and suitable marker which reflect the physiological exercise response and recovery processes in athletes’ during endurance exercises. IL-10 is primarily expressed by monocytes and represents an anti-inflammatory cytokine, while IL-15 is a regulator of the activity of T cells and natural killer cells^26^. Limitations in the methodological analysis of IL-10 and IL-15 are suggested to be the reason for the weak reliabilities within these cytokines. The results of both markers are below the limit of detection of all commercially available enzyme-linked immunosorbent assay (ELISA) kits. Thus, IL-10 and IL-15 are not seriously quantifiable by this method.

CK is a frequently used diagnostic marker for detecting exercise-induced muscle disruption or increased cell permeability. With regard to the assessment of suitability, CK is exercise sensitive and CK blood concentrations highly correlated to MVC values at different time points. Contrary to our expectations, for CK, only a moderate reliability was found. Due to the high interindividual variability in serum CK, the assignment of reliable reference values for athletes is complicated^10^. In accordance with these results, a study of Roe et al. (2016) found only a low reliability with a high coefficient of variation and a poor sensitivity measured in rugby players^27^. It is suggested that variabilities in CK release after exercise is caused by the existence of high and low responders due to the availability of different gene polymorphisms. However, other factors, such as training status or gender, might affect the reliability of CK^9^. In addition, the peak of CK values was somewhat delayed at 24 h after the RFTs, making it difficult to associate this parameter to muscular fatigue and to represent possible muscular recovery processes. This finding is consistent with other data that proved a poor correlation of CK values with other objective physiological markers^25^. However, it should be evaluated if CK is a more eligible marker for determination of muscular damage after eccentric or unaccustomed exercise over days.

For the majority of the haematological markers, a high reliability was found. These parameters include MCV, HGB, PLT, RBC, HCT, and MCHC. Excellent reliability was calculated for MCV. MCV is a predictive indicator for haematological diseases and according to findings of the resulting data, also a suitable marker to represent the physiological exercise response and recovery processes. A review of seasonal variations of haematological parameters in athletes summarizes concordant characteristics within the same sport discipline^28^. It could conceivably be hypothesized that the methodological analyses of haematological markers have a higher stability compared to the cytokine or enzyme measurements. It is assumed that the immediate processing and the lack of any centrifugation or freezing procedures result in a higher reliability. However, a previous study has proven a potential negative impact on the stability of cytokine measurements in plasma^29^. While haematological markers might be affected by an increased mechanotransduction and plasma volume contraction during strenuous endurance exercise bouts^30^, most cytokines are released by different cell types and are involved in multiple physiological processes. However, data about the reliability of haematological markers are rare, although many studies have called for follow-up studies to review the quality criteria^31^.

Interestingly, MVCs can be classified as highly reliable. In line with previous studies, a decreased MVC of knee extension, as well as a reduction in MVC of knee flexion, were found after endurance exercise trials^18^. Similarly, an excellent reliability of MVC was previously confirmed^32^. Previous studies which quantify the load condition after endurance exercise use the MVCs of the lower limbs to examine muscular fatigue directly by functional testing^18^. Thus, MVC analysis of knee flexion and knee extension might be useful functional tests to analyze exercise load and recovery in endurance exercise.

It is somewhat surprising that the MDMQ data quoted higher reliabilities compared to Borg scale (BS) values. RPE is regularly used in sports science research and a valid parameter of internal training load^33^. However, not much is known about its reliability in the context of endurance exercise. In contrast, the score of multidimensional mood state is rarely used in sports science. Nevertheless, a reliability of ICC = 0.70 was found, indicating that the questionnaire might be a suitable complementary diagnostic tool for the assessment of exercise load and recovery processes in endurance sports.

Interestingly, the training status had negligible effects on the physiological exercise response. Accordingly, the suitability of the exercise response and recovery markers was not significantly different between the subgroups of trained and untrained individuals. However, the reason might be that we did not recruit highly trained endurance athletes for this study. Surprisingly, we found a difference in the reliability of CK and CRP. Here, CK showed a lower reliability for trained compared to untrained subjects, while for CRP higher reliability was found in the trained subgroup.

Finally, a number of important limitations need to be considered. The preliminary testing, as well as further exercise trials in the study, were performed in the field rather than under laboratory conditions. In addition, we did not use the maximum oxygen consumption (VO_2max_) as a gold standard to control exercise intensities^34^. Therein, we see greater benefits from our method, particularly regarding the transferability in sports practice. Due to the limited space, we could not analyze the differences between gender, menstruation cycle and training status. This study focused exclusively on research of suitable and reliable physiological exercise response and recovery markers with a high number of random participants.

In conclusion, the best reliability and suitability were found for TBARS and IL-1RA suggesting their eligibility as markers for the monitoring of exercise response and recovery processes in endurance sports. Also, a good reliability was found for LDH, while CK showed good suitability but only moderate reliability. Accordingly, data indicate to expand the panel of blood biomarkers to monitor the athletes’ load-recovery status in a reliable way. Perhaps, the use of a combination of selected blood markers, MVC measurements, and subjective assessment tools such as MDMQ, should be discussed. Further research in this field is needed to evaluate the suitability of marker combinations, which comprehensively assess physiological exercise response and recovery processes in athletes. These findings should be combined with the development of innovative analyzing tools, that can be applied by athletes and trainers.

## Methods

### Subjects

For a test–retest study design, 106 trained and untrained male and female subjects, aged 19–43 years, were recruited randomly and voluntarily to participate. 62 (31 male and 31 female) of them completed all examinations and were included in statistical analysis. According to the American College of Sports Medicine guidelines for exercise testing and prescription, the participants were differentiated based on their endurance capacity in subgroups of trained (T) (N = 37) and untrained (UT) (N = 25) individuals^35^. The T group consisted mainly of runners, strength athletes, and semi-professional team sports players such as soccer, handball, and volleyball players. All other subjects were defined as either recreational active or inactive individuals. Their personal characteristics and anthropometric data are collectively shown in Table 1. All subjects were informed about the nature, purpose, and potential risks of the study and signed an informed consent statement prior to study participation. The local Ethical Committee of the Justus-Liebig-University Giessen (Germany) reviewed the study and approved ethical clearance, which was obtained according to the Declaration of Helsinki. In order to ensure that all subjects were physically healthy and fit enough to participate in sporting activities, they were medically screened. Exclusion criteria consisted of smoking, pregnancy, mothers in the lactation period, cardiovascular diseases, acute infections, musculoskeletal injuries, acute symptomatic respiratory deficits, and chronic diseases.

**Table 1:** Subject characteristics and anthropometric data (n = 62).

Variables	Overall	Trained (T)	Untrained (UT)
Age (years)	25.3 ± 4.6 (19–43)	25.62 ± 5.3 (19–43)	24.8 ± 3.4 (19–35)
Height (cm)	175.8 ± 10.1 (155–201)	172.2 ± 8.8 (155–191.5)	181.2 ± 9.5 (158–201)
Weight (kg)	72.9 ± 13.8 (50–109)	67.8 ± 10.7 (50–99)	80.6 ± 14.6 (52.5–109)
BMI* (kg/m^2^)	23.4 ± 2.8 (18.8–30.2)	22.8 ± 2.5 (18.8–28.8)	24.4 ± 3.1 (19.9–30.2)

Data are presented as Mean ± SD (Min.–Max.).

*BMI body mass index.

### Experimental approach: preliminary testing

The first step of the experimental approach contains testing of endurance capacity parameters to monitor the kinetics of various markers during further two identical strenuous exercise trials under controlled conditions. Subjects were tested for their endurance capacity during a continuous progressive exercise field test using lactate diagnostic, as previously described^36^. Briefly, subjects started on a 200 m running track at 6 km/h and increased their running speed by 2 km/h every three minutes until subjective exhaustion. Prior to the field test, between the three-minute stages with a break of 30 s and immediately after exhaustion, 20 µL of capillary blood was taken from the earlobe with an end-to-end glass capillary. Heart rate (HR) was continuously tracked using HR monitors (Polar FT1, Polar Electro Oy, Finland). Blood lactate values were analyzed using enzymatic-amperometrical detection (Bosen S-Line Plus, EKF-Diagnostics Sales GmbH, Magdeburg, Germany). HR and blood lactate values were used to evaluate the individual anaerobic threshold (IAT) using the Ergonizer Software for medical application (Ergonizer Software 4.9.4, Freiburg, Germany). The IAT was used to determine the individual running intensity during the following strenuous exercise trials. Calculation of IAT was performed by adding the constant value of 1.5 mmol/L to lactate concentration at the individual's lactate threshold^37^.

### Experimental approach: testing days of strenuous exercise trials

Approximately one week after the preliminary test, first testing day TD1 of strenuous exercise trial took place. Both testing days (TDs) started between 8:00 and 9:00 am for each subject. Prior to the TDs, subjects were instructed on several standardized conditions to which they had to comply. From four days before the particular TDs, subjects were not allowed to take part in any exhausting physical activity, only regenerative training was acceptable. Furthermore, it was forbidden to consume alcohol the day before. A nutrition protocol had to be drawn up, which included all consumed drinks and meals one day prior TD1 as well as breakfast on the testing day. The protocol served as a guideline for the food intake prior to the second testing day (TD2) to ensure standardized conditions. At the respective testing day, subjects had to fill out a questionnaire concerning their regular physical activity and their usual nutrition. All participants did not change their regularities in nutrition as well as in physical activity in between the exercise trials. In female subjects, the menstrual cycle was documented as well. These questionnaires were issued to document large deviations in these habits and to exclude possible changes in physical performance between TD1 and TD2.

The testing procedure contains two identical 60-min continuous endurance running field tests RFTs, intermitted by a recovery period of approximately four weeks. In order to examine the test–retest reliability of measured parameters, the previously described standardized conditions during both TDs were given high significance. The exercise protocol consisted of 40 min running at an intensity corresponding to 95% of HR at IAT, followed by 20 min at 110% in order to ensure exhaustion. The participants completed both RFTs at the same duration at the respective HR. Specific duration and intensity were chosen after the evaluation of a pilot study as well as in previous studies^38^. The outcome measures were collected before, immediately after, 3 and 24 h after each exercise test by double analysis. MTPs were chosen to make data comparable to previous studies^11,21^.

### Blood biomarkers

Venous blood samples were collected in vacutainers. Plasma vacutainers were anticoagulated with EDTA. It was centrifuged at 2,500 × g for 10 min at 4 °C immediately after sampling, while serum samples had clotted for 30 min before centrifugation. Samples were separated into aliquots and stored in Eppendorf tubes at −80 °C until analysis. IL-1RA, IL-8, IL-15 and IL-10 were determined by high-sensitivity ELISA (Quantikine ELISA Kits: R&D Systems, analytical sensitivity: IL-1RA: 18.3 pg/mL, IL-8: 0.4 pg/mL, IL-10: 0.17 pg/mL, IL-15: 2 pg/mL; MVZ, Koblenz, Germany). Enzymes, CK and LDH, as well as the plasma protein CRP were analyzed by ELISA using a Cobas 8,000 Immunoassay System (Roche Diagnostics, analytical sensitivity: CK: 7 U/L, LDH: 10 U/L, CRP: 0.3 mg/L; MVZ, Koblenz, Germany). Levels of cortisol and IL-6 were measured by Advia Centaur XPT immunoassay system (Siemens, analytical sensitivity: cortisol: 3 ng/mL, IL-6: 2.7 pg/mL; MVZ, Koblenz, Germany). Plasma concentration of TBARS, a metabolite of lipid peroxidation, was determined spectrofluorimetrically. Briefly, plasma samples were heated with thiobarbituric acid reagent at 100 °C for 60 min. After cooling, it was neutralized with alkaline methanol. Finally, samples were centrifuged at 3,000 × g and TBARS levels were measured by fluorescence signals (excitation wavelength 532 nm; emission wavelength 553 nm; Fluorescence Spectrometer LS55, PerkinElmer, Rodgau, Germany). Numbers of WBC, RBC, LYM, PLT, and NEU were determined using an automated haematology analyzer (Sysmex KX-21 N Autoanalyzer, TOA Electronics, Japan). Furthermore, levels of HGB, HCT, MCH, MCV and MCHC were evaluated. SII, PLR as well as NLR were calculated as previously described^39^.

### Muscle force parameters

To investigate the muscle force performance of the lower limbs, a 5-bound test (5BT) for measuring jump distance and an isometric strength test for measuring maximum voluntary contraction of knee flexion and extension were used. The 5BT was carried out as previously described^40^. Briefly, subjects were required to stand with their preferred foot forward at a marked starting point and bound five consecutive bounds with alternating left and right foot. Three trials were performed and the jump with the largest horizontal distance (m) was documented. The jump distance was measured from the marked starting position to the heel of the rear foot after the fifth jump. MVC of knee extensors and knee flexors were analyzed using isometric strength dynamometer m3diagnos (Schnell, Peutenhausen, Germany). First, subjects were seated and fixed in a standardized position with a defined device angle of 60° to measure the MVC of the knee extensors. An abdomen belt and crossed arms in front of the chest limited any extraneous movements of the upper body. Secondly, the MVC of knee flexors was examined in a lying position with a defined device angle of 150°. For each test, the best value of two trials was recorded. MVCs were calculated by analysis software (Diagnos Professional V1.0, Schnell).

### Subjective parameters

The subjective RPE were requested using the BS^41^. Furthermore, each subject had to complete a German version of the MDMQ^42^. It contains twelve items rated on a five-point Likert scale and measures three subscales (good-bad mood, alertness-tiredness and calmness-restlessness). These subscales are summed up, yielding a score between four and 20, with higher scores indicating better mood, higher alertness and calmness. From all three subscales, an index between twelve and 60 was calculated, which reflects the acute multidimensional mood state.

### Statistics

Data of all subjects are presented as means ± standard deviation of the mean and the minimum and maximum values. In cases of normal or log-normal distribution (Kolmogorov–Smirnov test), data were analyzed using the two-way ANOVA to observe mean differences between the MTPs depending on the TDs. If analysis revealed any significant main effects between the MTPs (*p* < 0.05), post hoc analysis was conducted by using the Bonferroni test. To consider training status, we separated the participants into subgroups of trained and untrained individuals and added these as between-subjects factor into the ANOVA analysis. Furthermore, analysis of the test–retest reliability between the MTPs of TD1 and TD2 was carried out with the ICC (model: two-way mixed effects; type: single measurement; definition: absolute agreement). In all cases, ICC > 0.5 was accepted as a minimal test–retest reliability. Values between 0.5 and 0.75, 0.75 and 0.9, and greater than 0.9 are indicative of moderate, good, and excellent reliability^43^. Pearson’s correlation analysis was used to analyze the suitability of the parameters. In all cases, *p* < 0.05 was accepted as being significant. Statistical power analysis was performed according Cohen et al. (1988)^44^. All statistical analyses were carried out using SPSS version 25 (IBM SPSS Statistics 25, IBM GmbH, Munich, Germany). Figures were created with GraphPad Prism 5.01.

## Data Availability

The datasets generated during and/or analysed during the current study are available from the corresponding author (Karsten Krüger; Karsten.Krueger@sportwiss.uni-giessen.de) on reasonable request and with permission from all involved institutions.

## References

[1] Halson SL (2014). Monitoring training load to understand fatigue in athletes. Sports Med. (Auckland, N. Z.).

[2] Kellmann M (2018). Recovery and performance in sport: consensus statement. Int. J. Sports Physiol. Perf..

[3] Thorpe RT, Atkinson G, Drust B, Gregson W (2017). Monitoring fatigue status in elite team-sport athletes: implications for practice. Int. J. Sports Physiol. Perf..

[4] Strimbu K, Tavel JA (2010). What are biomarkers?. Curr. Opin. HIV AIDS.

[5] Finsterer J, Drory VE (2016). Wet, volatile, and dry biomarkers of exercise-induced muscle fatigue. BMC Musculoskelet. Disord..

[6] Cristalli DO, Arnal N, Marra FA, de Alaniz MJ, Marra CA (2012). Peripheral markers in neurodegenerative patients and their first-degree relatives. J. Neurol. Sci..

[7] Lee EC (2017). Biomarkers in sports and exercise: tracking health, performance, and recovery in athletes. J. Strength Conditioning Res..

[8] Smith LL (2000). Cytokine hypothesis of overtraining: a physiological adaptation to excessive stress?. Med. Sci. Sports Exerc..

[9] Brancaccio P, Maffulli N, Limongelli FM (2007). Creatine kinase monitoring in sport medicine. Br. Med. Bull..

[10] Meyer T, Kellmann M, Ferrauti A, Pfeiffer M, Faude O (2013). The measurement of recovery and regeneration requirements in football. Ger. J. Sports Med..

[11] Schild M (2016). Effects of acute endurance exercise on plasma protein profiles of endurance-trained and untrained individuals over time. Mediators Inflamm..

[12] Romagnoli M (2014). Influence of training and a maximal exercise test in analytical variability of muscular, hepatic, and cardiovascular biochemical variables. Scand. J. Clin. Lab. Invest..

[13] Theofilidis G, Bogdanis GC, Koutedakis Y, Karatzaferi C (2018). Monitoring exercise-induced muscle fatigue and adaptations: making sense of popular or emerging indices and biomarkers. Sports (Basel, Switzerland).

[14] Proschinger S, Freese J (2019). Neuroimmunological and neuroenergetic aspects in exercise-induced fatigue. Exerc. Immunol. Rev..

[15] Hecksteden A (2016). Blood-borne markers of fatigue in competitive athletes: results from simulated training camps. PLoS ONE.

[16] Stenholm S (2010). Anabolic and catabolic biomarkers as predictors of muscle strength decline: the InCHIANTI study. Rejuven. Res..

[17] Pedlar CR, Newell J, Lewis NA (2019). Blood biomarker profiling and monitoring for high-performance physiology and nutrition: current perspectives, limitations and recommendations. Sports Med. (Auckland, N. Z.).

[18] Presland JD, Dowson MN, Cairns SP (2005). Changes of motor drive, cortical arousal and perceived exertion following prolonged cycling to exhaustion. Eur. J. Appl. Physiol..

[19] Lattier G, Millet GY, Martin A, Martin V (2004). Fatigue and recovery after high-intensity exercise part I: neuromuscular fatigue. Int. J. Sports Med..

[20] Ament W, Verkerke GJ (2009). Exercise and fatigue. Sports Med. (Auckland, N. Z.).

[21] Kruger K (2016). Apoptosis of T-cell subsets after acute high-intensity interval exercise. Med. Sci. Sports Exerc..

[22] Mohr M (2016). Muscle damage, inflammatory, immune and performance responses to three football games in 1 week in competitive male players. Eur. J. Appl. Physiol..

[23] Cobley JN, Close GL, Bailey DM, Davison GW (2017). Exercise redox biochemistry: conceptual, methodological and technical recommendations. Redox Biol.

[24] Del Coso J (2013). Running pace decrease during a marathon is positively related to blood markers of muscle damage. PLoS ONE.

[25] Scott JP (2013). Cytokine response to acute running in recreationally-active and endurance-trained men. Eur. J. Appl. Physiol..

[26] Clark SE, Burrack KS, Jameson SC, Hamilton SE, Lenz LL (2019). NK cell IL-10 production requires IL-15 and IL-10 driven STAT3 activation. Front Immunol.

[27] Roe G (2016). Between-days reliability and sensitivity of common fatigue measures in rugby players. Int. J. Sports Physiol. Perf..

[28] Banfi G, Lundby C, Robach P, Lippi G (2011). Seasonal variations of haematological parameters in athletes. Eur. J. Appl. Physiol..

[29] Huang WY (2017). Impact of freeze-thaw cycles on circulating inflammation marker measurements. Cytokine.

[30] Wang JS (2004). Intense exercise increases shear-induced platelet aggregation in men through enhancement of von Willbrand factor binding, glycoprotein IIb/IIIa activation, and P-selectin expression on platelets. Eur. J. Appl. Physiol..

[31] Alis R, Sanchis-Gomar F, Risso-Ballester J, Blesa JR, Romagnoli M (2016). Effect of training status on the changes in platelet parameters induced by short-duration exhaustive exercise. Platelets.

[32] De Ruiter CJ, Hamacher P, Wolfs BG (2016). A short submaximal test to determine the fatigue threshold of knee extensors in young men. Med. Sci. Sports Exerc..

[33] Mann RH, Williams CA, Clift BC, Barker AR (2019). The validation of session rating of perceived exertion for quantifying internal training load in adolescent distance runners. Int. J. Sports Physiol. Perf..

[34] Carlson DJ (1995). VO2max: the gold standard?. Chest.

[35] Riebe D, Ehrman JK, Liguori G, Magal M, Medicine ACOS (2018). ACSM's guidelines for exercise testing and prescription.

[36] Kruger K, Pilat C, Uckert K, Frech T, Mooren FC (2014). Physical performance profile of handball players is related to playing position and playing class. J. Strength Cond. Res..

[37] Kindermann W, Simon G, Keul J (1979). The significance of the aerobic-anaerobic transition for the determination of work load intensities during endurance training. Eur. J. Appl. Physiol..

[38] Haller N, Tug S, Breitbach S, Jorgensen A, Simon P (2017). Increases in circulating cell-free DNA during aerobic running depend on intensity and duration. Int. J. Sports Physiol. Perf..

[39] Fest J (2018). Reference values for white blood-cell-based inflammatory markers in the Rotterdam Study: a population-based prospective cohort study. Sci. Reports.

[40] Coutts AJ, Slattery KM, Wallace LK (2007). Practical tests for monitoring performance, fatigue and recovery in triathletes. J. Sci. Med. Sport.

[41] Borg GA (1982). Psychophysical bases of perceived exertion. Med. Sci. Sports Exerc..

[42] Steyer R, Schwenkmezger P, Notz P, Eid M (1997). Multidimensional mood state questionnaire (MDBF).

[43] Koo TK, Li MY (2016). A guideline of selecting and reporting intraclass correlation coefficients for reliability research. J. Chiropract. Med..

[44] Cohen J (1988). Statistical power analysis for the behavioral sciences.

